# *Pmorf0222*, a Virulence Factor in Pasteurella multocida, Activates Nuclear Factor Kappa B and Mitogen-Activated Protein Kinase via Toll-Like Receptor 1/2

**DOI:** 10.1128/iai.00193-22

**Published:** 2022-12-21

**Authors:** Tenglin Xu, Yating Zheng, Benjin Liu, Mingxing Kou, Qian Jiang, Jiasen Liu, Hongtao Kang, Mingfa Yang, Dongchun Guo, Liandong Qu

**Affiliations:** a Division of Zoonosis of Natural Foci, State Key Laboratory of Veterinary Biotechnology, Harbin Veterinary Research Institute, Chinese Academy of Agricultural Sciences, Harbin, People’s Republic of China; University of Pennsylvania

**Keywords:** *Pasteurella multocida*, virulence factor, TLR1/2, MAPK/NF-κB, proinflammatory cytokine

## Abstract

Pasteurella multocida primarily causes hemorrhagic septicemia and pneumonia in poultry and livestock. Identification of the relevant virulence factors is therefore essential for understanding its pathogenicity. *Pmorf0222*, encoding the PM0222 protein, is located on a specific prophage island of the pathogenic strain C48-1 of P. multocida. Its role in the pathogenesis of P. multocida infection is still unknown. The proinflammatory cytokine plays an important role in P. multocida infection; therefore, murine peritoneal exudate macrophages were treated with the purified recombinant PM0222, which induced the secretion of tumor necrosis factor alpha (TNF-α) and interleukin-1β (IL-1β) via the Toll-like receptor 1/2 (TLR1/2)–nuclear factor kappa B (NF-κB)/mitogen-activated protein kinase (MAPK) signaling and inflammasome activation. Additionally, the mutant strain and complemented strain were evaluated in the mouse model with P. multocida infection, and PM0222 was identified as a virulence factor, which was secreted by outer membrane vesicles of P. multocida. Further results revealed that *Pmorf0222* affected the synthesis of the capsule, adhesion, serum sensitivity, and biofilm formation. Thus, we identified *Pmorf0222* as a novel virulence factor in the C48-1 strain of P. multocida, explaining the high pathogenicity of this pathogenic strain.

## INTRODUCTION

Pasteurella multocida is a pathogen infecting both domestic and wild animals, mainly causing diseases such as fowl cholera in avian species, bovine hemorrhagic septicemia in ruminant species, progressive atrophic rhinitis and pneumonic pasteurellosis in swine, and snuffles in rabbits (1, 2). P. multocida is often recovered from humans with wound abscesses or meningitis following cat or dog bites (2). P. multocida strains are classified into serogroups A, B, D, E, and F based on the type specificity of the capsule (3, 4). Although P. multocida has been known for more than 100 years, its pathogenesis and the host-pathogen interactions are relatively unknown.

The stomatins, prohibitins, flotillins, and HflK/C (SPFH) proteins with a conserved prohibitin (PHB) or SPFH domain, which is widely distributed in archaea, bacteria, and eukaryotes, are named the SPFH superfamily proteins (5). The SPFH domain proteins form high-ordered homo-oligomeric complexes that are localized in microdomains, which are essential for the formation and maintenance of the correct function of the prokaryotic lipid rafts (6, 7). The flotillin homologue FloT/A in Bacillus subtilis is essential for the kinase PhoR/P and ResE/D two-component signal transduction system participating in the formation of the bacterial functional membrane microdomains (FMMs) (8). The FMMs in either eukaryotic cells or bacteria are referred to as lipid rafts. These microdomains are associated with various cellular processes, such as formation of the outer membrane vesicles (OMVs), protein secretion, signal transduction, protein degradation or sorting, biofilm formation, and pathogen invasion (8, 9). The decreased expression of flotillin-1/reggie-2 has been reported to reduce the epidermal growth factor (EGF)-induced phosphorylation of the specific tyrosinase in the EGF receptor, inefficiently activate the downstream mitogen-activated protein kinase (MAPK) and Akt signaling, and directly inactivate the extracellular-regulated kinase 1/2 (ERK1/2) (10).

Following infection with P. multocida, multiple organs such as the lungs, liver, and spleen of various poultry often exhibit pathological defects, including inflammation, necrosis, and multiple hemorrhages on the serosal surfaces (1). The molecular basis of the pathogenesis of P. multocida mainly involves virulence factors such as the capsule, lipopolysaccharide (LPS), flagella, adhesin, P. multocida toxin (PMT), and outer membrane proteins (4). These virulence factors are essential for adherence, invasion, and resistance to killing by the host immune system. In addition, recent studies indicated that these factors can induce inflammatory reactions and cause severe inflammation in diseases (11–13). For example, PM0442, a lipoprotein located at the outer membrane of P. multocida, can induce inflammatory responses via Toll-like receptor 2 (TLR2) and NF-κB/MAPK signaling pathways (13). The PMT protein can induce proinflammatory cytokines tumor necrosis factor alpha (TNF-α), interleukin-1β (IL-1β), and IL-6 via the G-protein-coupled receptor and JAK-STAT signaling pathway (14). In our previous study, we predicted that the *Pmorf0222* gene encoding the PM0222 protein is an HflC/K-like protein, which is a non-phage-related protein in the lysogenic phage gene cluster of the pathogenic strain C48-1 of P. multocida (15). In this study, we investigated the roles of *Pmorf0222* in the pathogenicity of P. multocida strain C48-1 and in the secretion of proinflammatory cytokines by macrophages.

## RESULTS

### The recombinant PM0222 protein induces proinflammatory cytokine secretion by murine peritoneal exudate macrophages.

**FIG 1:**
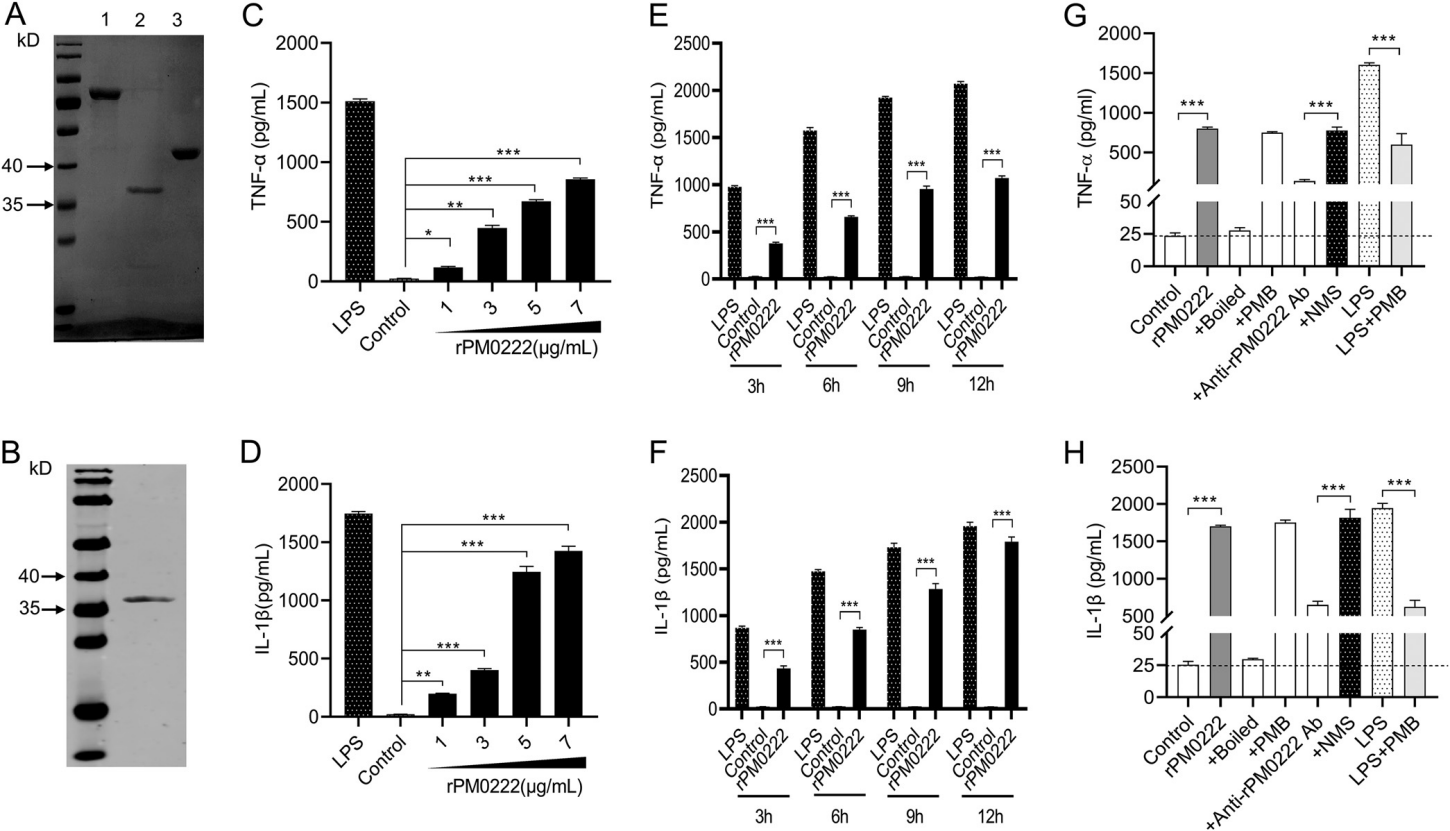
rPM0222-induced secretion of proinflammatory cytokines by murine peritoneal exudate macrophages. (A) Lane 1, purification of recombinant MBP-PM0222 fusion protein. Lane 2, purification of rPM0222 protein. Lane 3, purification of MBP. (B) rPM0222 reacted with the anti-His antibody. (C and D) The murine peritoneal exudate macrophages were incubated with 5 μg/mL rPM0222 (1, 3, 5, or 7 μg/mL) for 6 h. The TNF-α and IL-1β levels in cell culture supernatants were measured using enzyme-linked immunosorbent assay (ELISA). (E and F) The murine peritoneal exudate macrophages were incubated with 5 μg/mL rPM0222, and the TNF-α and IL-1β levels in cell culture supernatants were measured using ELISA at 3, 6, 9, or 12 h. (G and H) The murine peritoneal exudate macrophages were incubated with 5 μg/mL rPM0222 for 6 h, and the TNF-α and IL-1β levels in cell culture supernatants were measured using ELISA. rPM0222 was pretreated with polymyxin B (+PMB) and boiled (+boiled) at 100°C for 10 min to confirm that the activation of murine peritoneal exudate macrophages was because of rPM0222 and not lipopolysaccharide (LPS). LPS (1 μg/mL) was used as a positive control and pretreated with polymyxin B (PMB) to confirm the action of PMB (LPS+PMB). To confirm that rPM0222 specifically promotes the secretion of proinflammatory cytokines, the proteins (5 μg/mL) were pretreated with the anti-rPM0222 serum (+anti-rPM0222 Ab, 1:500) or control mouse serum (+NMS, 1:500). Data are expressed as the mean ± standard deviation (SD) from three independent experiments. *, *P* < 0.05; **, *P* < 0.01; ***, *P* < 0.001. The experiments were performed at least three times.

PM0222 was identified as an HflK/C-like protein (5) using the Conserved Domain Architecture Retrieval Tool (https://www.ncbi.nlm.nih.gov/Structure/lexington/lexington.cgi?cmd=rps) (see Fig. S1A and Table S1 in the supplemental material). To investigate the role of the recombinant PM0222 (rPM0222) protein in inducing proinflammatory cytokines, rPM0222 was expressed and purified (Fig. 1A and B). From the fitting curve, we extrapolated that 5 μg rPM0222 protein was equivalent to the amount of PM0222 protein expressed by 1.3 × 10^8^ CFU of wild-type strain C48-1 (Fig. S1B and C). After the murine peritoneal exudate macrophages were stimulated with rPM0222 for 6 h, TNF-α and IL-1β were induced in a time- and dose-dependent manner (Fig. 1C to F and Fig. S2A to D). The induction could be blocked by anti-rPM0222 serum (Fig. 1G and H and Fig. S2E and F). These results indicated that rPM0222 could induce the secretion of proinflammatory cytokines TNF-α and IL-1β by murine peritoneal exudate macrophages.

### rPM0222 activates both TLR1/2–NF-κB/MAPK and inflammasome signaling pathways.

**FIG 2:**
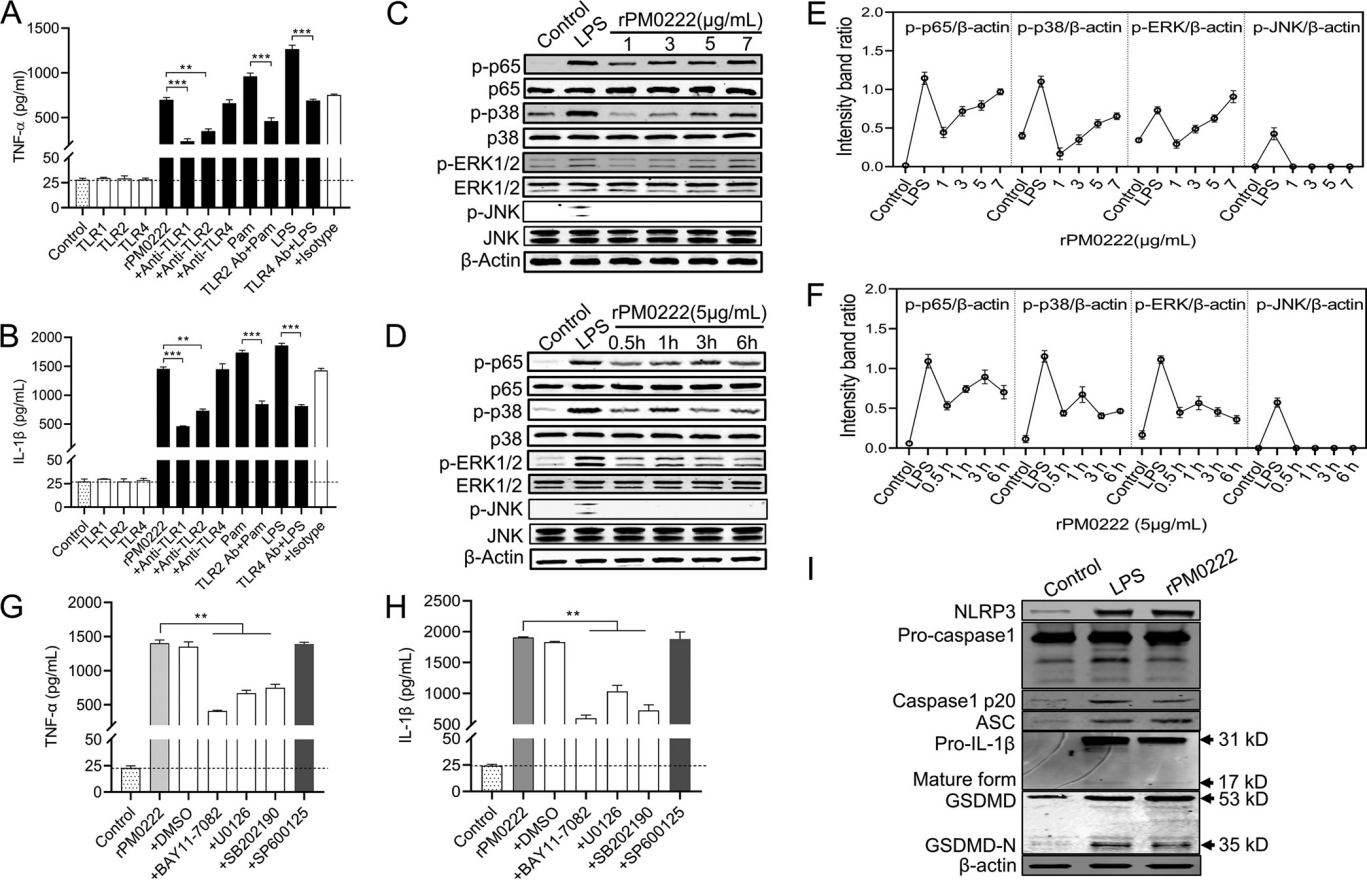
Secretion of TNF-α and IL-1β in response to rPM0222 is dependent on TLR1/2 and NF-κB/MAPK signaling. (A and B) The cells were pretreated with 5 μg/mL of anti-TLR1, -TLR2, and -TLR4 or an IgG isotype-matched control antibody for 1 h, followed by stimulation with rPM0222 (5 μg/mL) for 6 h. The cell culture supernatants were collected, and the levels of TNF-α and IL-1β were measured using ELISA. Pam, Pam3CSK4. (C and D) The murine peritoneal exudate macrophages were incubated with rPM0222 (1, 3, 5, or 7 μg/mL) for 6 h or incubated with rPM0222 (5 μg/mL) for the indicated times (0.5, 1, 3, or 6 h). Cell lysates were prepared, and the phosphorylation/activation status of p65, ERK1/2, p38, and JNK was analyzed using Western blotting. (E and F) The murine peritoneal exudate macrophages were incubated with rPM0222 (1, 3, 5, or 7 μg/mL) for 6 h or 5 μg/mL rPM0222 for the indicated times (0.5, 1, 3, or 6 h). The intensity band ratio of activation status of p65, ERK1/2, p38, and JNK to that of β-actin was analyzed. (G and H) The murine peritoneal exudate macrophages were pretreated with U0126 (+U0126; 20 μM), SB203580 (+SB202190; 20 μM), SP600125 (+SP600125; 20 μM), BAY11-7082 (+BAY11-7082; 20 μM), or dimethyl sulfoxide (0.01%) (+DMSO) for 1 h, followed by stimulation with rPM0222 (5 μg/mL) for 6 h. Further, the TNF-α and IL-1β levels in cell culture supernatants were measured using ELISA. (I) The murine peritoneal exudate macrophages were incubated with 5 μg/mL rPM0222 for 6 h. Cell lysates were prepared, and the levels of NLRP3, ASC, procaspase-1, p20, mature IL-1β, GSDMD, and GSDMD-N were measured using Western blot analysis. Data are expressed as the mean ± standard deviation from three independent experiments. **, *P* < 0.01; ***, *P* < 0.001. The experiments were performed at least three times.

rPM0222 could induce TNF-α, promoting the identification of the signaling for this protein to induce the secretion of proinflammatory cytokines. The mRNAs of *TLR1* and *TLR2* but not those of *TLR4* were upregulated after stimulation of the murine peritoneal exudate macrophages with rPM0222 (Fig. S3A to C). In addition, expression of TNF-α and IL-1β was inhibited by specific antibodies against TLR1 and TLR2 but not those against TLR4 (Fig. 2A and B and Fig. S3D and E). However, the changes in TNF-α and IL-1β secretion by the murine peritoneal exudate macrophages after treatment with anti-TLR1, -TLR2, or -TLR4 and isotype antibodies were not significantly different. These results indicated that TNF-α and IL-1β production induced by rPM0222 was mediated via TLR1/2 signaling.

p-p65, p-p38, and p-ERK1/2, which are the downstream signaling molecules of the TLR, were detected in a time- and dose-dependent manner (Fig. 2C and D). The amount of p-p65 reached its peak at 3 h, whereas those of p-p38 and p-ERK1/2 reached their peak at 1 h (Fig. 2E and F). However, no effect was observed on p-JNK. This indicated that rPM0222 activated the NF-κB, p38, and ERK1/2 signaling pathways in the murine peritoneal exudate macrophages. Additionally, TNF-α and IL-1β expression was significantly inhibited by the inhibitors of p65 (BAY11-7082), p38 (SB202190), and ERK (U0126) but not by those of JNK (SP600125) (Fig. 2G and H and Fig. S3F and G). These results supported the inference that rPM0222 induced the secretion of TNF-α and IL-1β via the TLR1/2–NF-κB/MAPK signaling pathways.

rPM0222 could induce IL-1β secretion, which is dependent on the activation of inflammasomes. The murine peritoneal exudate macrophages were treated with rPM0222 to evaluate its role in the inflammasome activation. Cleaved caspase-1 (p20) and gasdermin D (GSDMD)-N levels were observed to be increased (Fig. 2I), indicating that rPM0222 could activate the inflammasome signaling. In addition, treatment with LPS or rPM0222 resulted in ballooning of the membrane compared with the control group (Fig. S4). These results suggested that inflammasomes were activated by rPM0222 in the murine peritoneal exudate macrophages, leading to IL-1β production.

### *Pmorf0222* attenuates the virulence of P. multocida in a mouse model.

**FIG 3:**
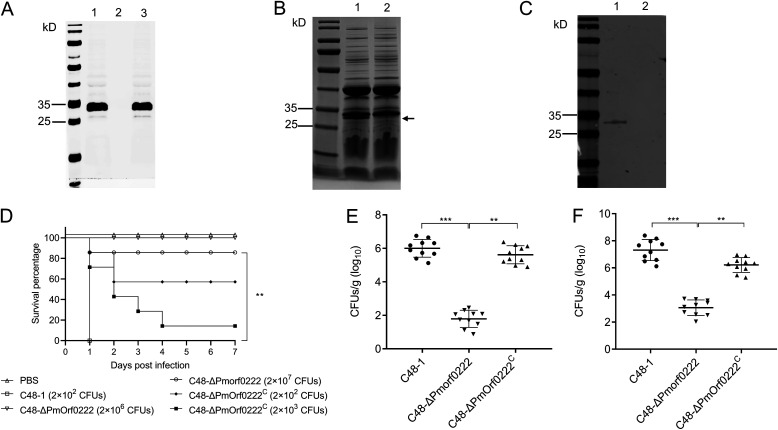
Assessment of the virulence of P. multocida in mice. (A) Confirmation of C48-Δ*Pmorf0222* mutant strain by Western blot analysis. The whole protein of the C48-1 wild-type strain (lane 1), C48-Δ*Pmorf0222* mutant strain (lane 2), and C48-Δ*Pmorf0222^C^* complemented strain (lane 3) was detected with anti-rPM0222 sera. (B) SDS-PAGE analysis of the outer membrane vesicles (OMVs) of P. multocida. Isolation of OMVs from wild-type strain (lane 1) and mutant strain (lane 2). (C) Total proteins of OMVs extracted from the wild-type strain and mutant strain reacted with the anti-rPM0222 sera as demonstrated by Western blot analysis. Isolation of OMVs from wild-type strain (lane 1) and mutant strain (lane 2). (D) Survival rates of each group of mice (*n *= 7) exposed to the wild-type strain, mutant strain, or complemented strain and sterile PBS (200 μL). (E and F) The bacterial load in each group of mice (*n *= 10) infected with the wild-type strain, mutant strain, or complemented strain in the liver (E) and spleen (F) 6 h postinfection. **, *P* < 0.01; ***, *P* < 0.001.

To evaluate the role of *Pmorf0222* in the virulence of P. multocida, the isogenic *Pmorf0222* mutant strain C48-Δ*Pmorf0222* was constructed based on the *Ng*Ago technology (Fig. 3A) and sequenced after 20 rounds of subculture (Fig. S5A). Further, the genetic stability of the C48-Δ*Pmorf0222* mutant strain was identified using PCR (Fig. S5B) and Western blot analysis (Fig. S5C). The recombinant plasmid pAL99-Pmorf0222 was electrotransformed into the mutant strain to obtain the complemented strain. Further, the C48-Δ*Pmorf0222* mutant strain exhibited a lower growth rate than the wild-type strain cells at 2 to 5 h (Fig. S5D). The isolated OMVs harbored a protein with a mass of 25 to 35 kDa that was deleted from the wild-type strain (Fig. 3B), which was confirmed using Western blot analysis (Fig. 3C).

When mice were infected with the wild-type strain C48-1 (2 × 10^2^ CFU/mouse), all mice died within 24 h. However, infection with the C48-Δ*Pmorf0222* mutant strain (2 × 10^2^ to 2 × 10^6^ CFU/mouse) did not cause any death during the trial until the 7th day postinfection; the survival percentage of the mice infected with the C48-Δ*Pmorf0222* mutant strain was significantly increased to 85.7% (2 × 10^7^ CFU/mouse), and those of the mice infected with the complemented strain C48-Δ*Pmorf0222^C^* were partially restored to 57.1% (2 × 10^2^ CFU/mouse) and 28.6% (2 × 10^3^ CFU/mouse) (Fig. 3D). Further, 6 h after the mice were infected, the proportion of C48-Δ*Pmorf0222* mutant bacterial colonies was observed to be significantly decreased in the liver (3.35-fold, *P* < 0.001) and spleen (2.39-fold, *P* < 0.001) of mice (Fig. 3E and F). The results suggested that lack of *Pmorf0222* could markedly attenuate the virulence of P. multocida strain C48-1.

### *Pmorf0222* promotes the production of capsular hyaluronic acid.

**FIG 4:**
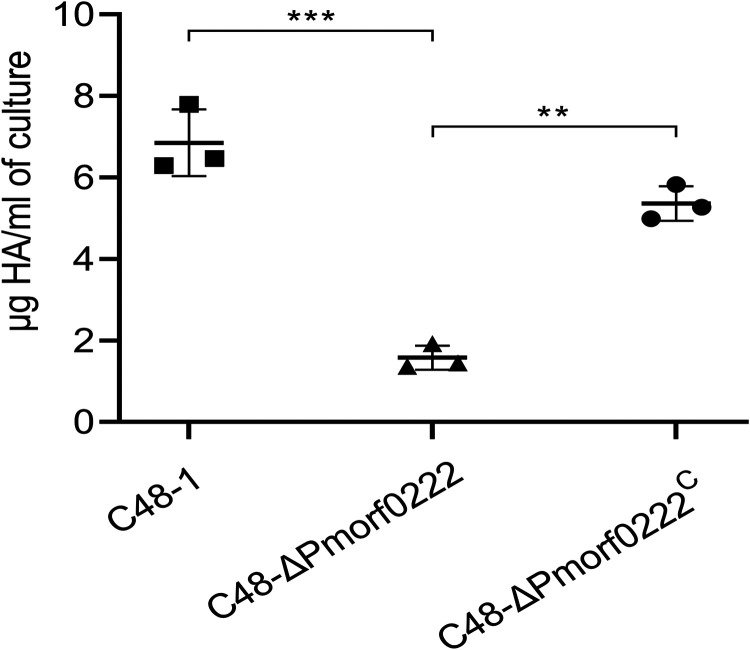
Detection of capsular hyaluronic acid (HA) production. The quantification of the hyaluronic acid content in the wild-type strain, mutant strain, and complemented strain at logarithmic phase (OD_600_ of approximately 0.6) with three replicates per group. Data are expressed as the mean ± standard deviation from three independent experiments. **, *P* < 0.01; ***, *P* < 0.001. The experiments were performed at least three times.

Previous reports indicated that the virulence of P. multocida was related to the production of the capsular hyaluronic acid (16, 17). To evaluate the role of *Pmorf0222* in the production of the capsular hyaluronic acid, its levels in the wild-type strain, mutant strain, and complemented strain were evaluated. Compared with the wild-type strain, the hyaluronic acid content in the mutant strain was observed to be reduced by 4.37-fold *in vitro* (*P* < 0.001), and in the complemented strain, the defective hyaluronic acid production could be partially restored (Fig. 4 and Fig. S6). These results indicated that *Pmorf0222* promoted the production of the capsular hyaluronic acid, which might be one of the factors for the contribution of *Pmorf0222* to virulence in P. multocida strain C48-1.

### *Pmorf0222* contributes to the adhesion to host cells.

**FIG 5:**
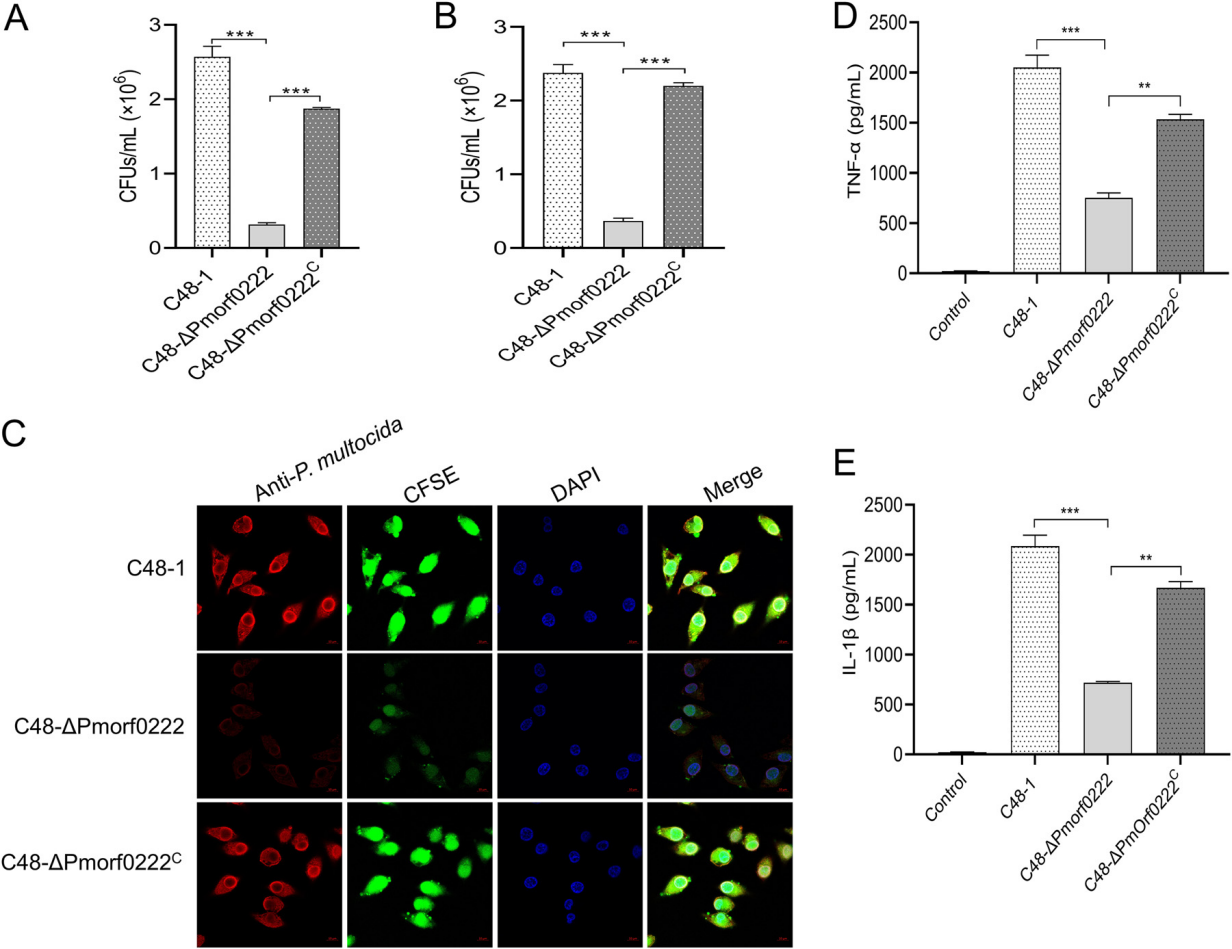
The ability of adhesion to the host cells by P. multocida. (A and B) Colony counts of the wild-type strain, mutant strain, or complemented strain infecting DF-1 (A) and HD11 (B) cells at a multiplicity of infection (MOI) of 10 for 2 h. (C) Detection of adhesion to HD11 cells by confocal laser scanning microscopy (CLSM; oil, ×100). HD11 cells were infected with the CFSE-labeled (green) wild-type strain, mutant strain, or complemented strain at an MOI of 10 for 2 h. Further, the CFSE-labeled wild-type strain, mutant strain, or complemented strain was recognized by antiserum against P. multocida C48-1 strains (red). Further, the infected HD11 cells were stained with DAPI (blue). (D and E) The murine peritoneal exudate macrophages were incubated with the wild-type strain, mutant strain, or complemented strain at an MOI of 10 for 6 h. The TNF-α and IL-1β levels in cell culture supernatants were measured using ELISA. Data are expressed as the mean ± standard deviation from three independent experiments. *, *P* < 0.05; **, *P* < 0.01; ***, *P* < 0.001. The experiments were performed at least three times.

Previous data suggested that capsular hyaluronic acid regulates the adherence of the P. multocida serogroup A to host cells (17). In this study, HD11 (chicken macrophage-like cell line) and DF-1 (chicken embryo fibroblast cell line) cells were inoculated with the wild-type strain, mutant strain, and complemented strain. Compared with the wild-type strain, the adhesion of the mutant strain to DF-1 and HD11 cells was observed to be significantly reduced by 8.11- (*P* < 0.001) and 6.48-fold (*P* < 0.001), respectively, 2 h postinfection (Fig. 5A and B). Subsequently, the colocalization of HD11 cells and bacteria was assessed using confocal laser scanning microscopy (CLSM). It was confirmed that in the mutant strain, the ability of the bacterial cells to adhere to HD11 cells was considerably reduced (Fig. 5C). Furthermore, a significant reduction was observed in IL-1β and TNF-α secretion by HD11 cells (Fig. S7A and B) and murine peritoneal exudate macrophages (Fig. 5D and E and Fig. S7C and D) exposed to the C48-Δ*Pmorf0222* mutant strain compared with those exposed to the wild-type strain and complemented strain. These results indicated that *Pmorf0222* might play an essential role in adhesion of P. multocida strain C48-1 to host cells and in the induction of proinflammatory cytokines.

### *Pmorf0222* enhances sensitivity to chicken serum.

**FIG 6:**
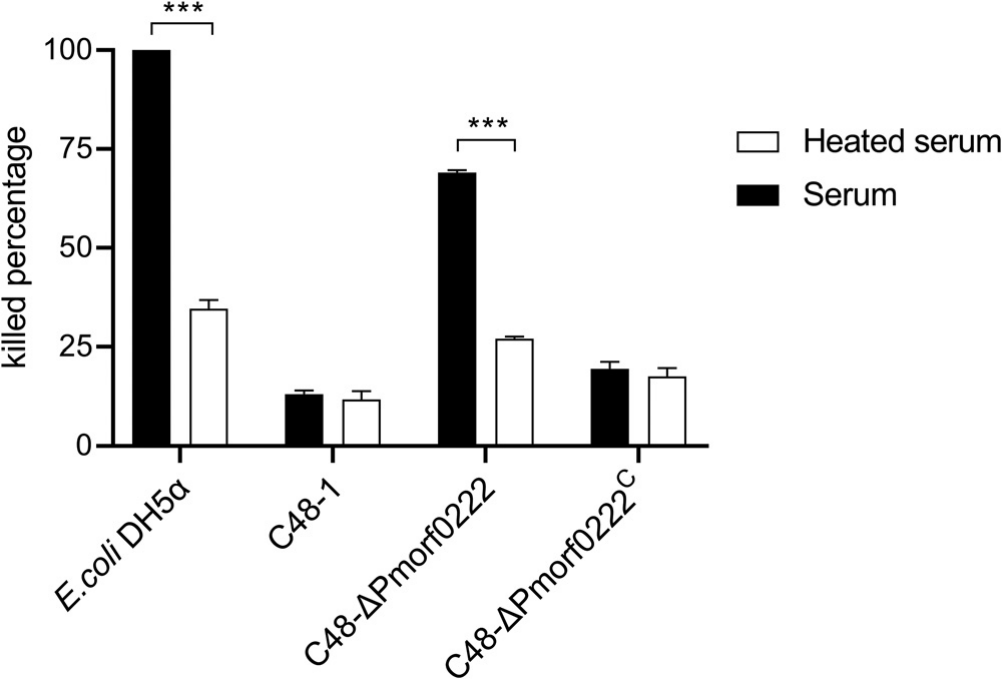
The sensitivity of P. multocida to chicken serum. The killed percentage of the wild-type strain, mutant strain, and complemented strain in the unheated and heated sera is given. When CFU/mL at 3 h < CFU/mL at 0 h, killed percentage = |(CFU/mL at 3 h) − (CFU/mL at 0 h)|/(CFU/mL at 0 h) × 100%. When CFU/mL at 3 h ≥ CFU/mL at 0 h, killed percentage = (CFU/mL at 0 h)/|(CFU/mL at 3 h) − (CFU/mL at 0 h)| × 100%. Data are expressed as the mean ± standard deviation from three independent experiments. ***, *P* < 0.001. The experiments were performed at least three times.

To determine serum sensitivity, the wild-type strain, mutant strain, and complemented strain were incubated with the specific-pathogen-free (SPF) chicken serum. The viability of the control strain Escherichia coli DH5α was 3.89-fold decreased in the heat-treated serum (*P* < 0.01) compared with that of E. coli DH5α in the unheated serum, which confirmed the bactericidal activity of serum. However, compared with the wild-type strain, the survival ratio of the mutant strain and complemented strain was observed to be reduced by approximately 28- (*P* < 0.001) and 1.41-fold, respectively, in the unheated serum (Table S3). Furthermore, between unheated and heated sera, the killed percentage of the mutant strain was observed to be significantly changed at 3 h, whereas that of the wild-type strain and complemented strain did not exhibit any change at 3 h (Fig. 6). These results indicated that the C48-Δ*Pmorf0222* strain was more sensitive to serum killing.

### *Pmorf0222* regulates the expression of other virulence genes.

**FIG 7:**
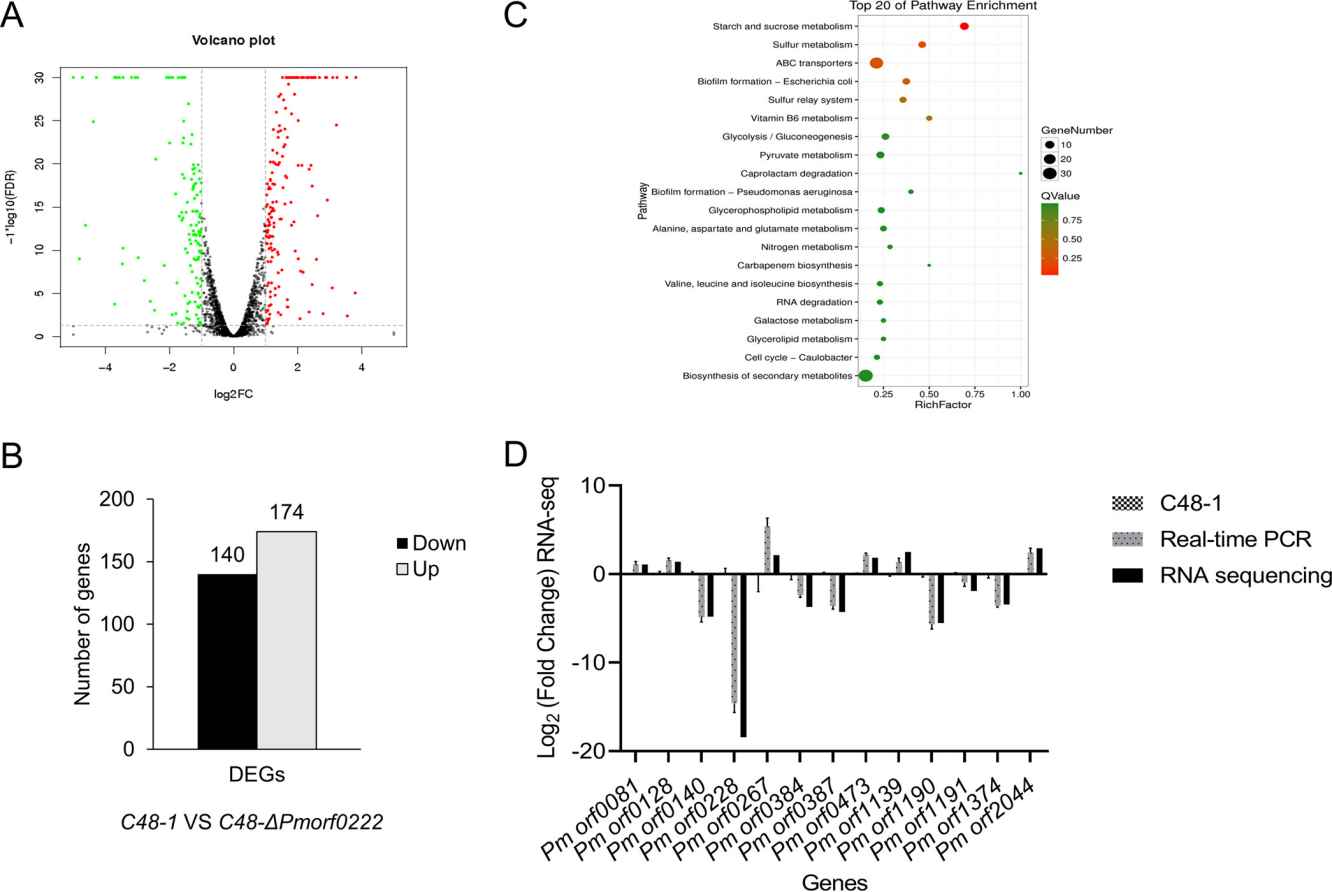
The expression of the virulence genes by transcriptome analysis. (A) Volcano plot for clustering of differentially expressed genes (DEGs; fold change [FC] of ≥1) of the wild-type strain and mutant strain. FDR, false-discovery rate. (B) The up- or downregulated DEGs (FC of ≥1) in the wild-type strain and mutant strain. (C) The top 20 enriched KEGG pathways for the up- or downregulated DEGs (FC of ≥1) in the wild-type strain and mutant strain. (D) To validate the reliability of the data obtained by transcription *in vitro*, 13 DEGs obtained after analyzing the RNA sequence were validated using qRT-PCR. The experiments were performed at least three times. RNA-seq, RNA sequencing.

The differentially expressed genes (DEGs) between the wild-type strain and mutant strain were compared using transcriptome analysis *in vitro* to determine the effects of *Pmorf0222* on other virulence genes in P. multocida. A total of 314 DEGs (fold change of ≥1) were enriched, containing 174 upregulated and 140 downregulated genes (Fig. 7A and B). The Kyoto Encyclopedia of Genes and Genomes (KEGG) pathway analysis revealed that the DEGs were predominantly enriched in the ABC transporters, starch and sucrose metabolism, biofilm formation, and biosynthesis of secondary metabolites (Fig. 7C). Overall, 13 DEGs obtained from the RNA sequence analysis were validated based on the reference gene *gyrB* using quantitative real-time PCR (qRT-PCR) (Fig. 7D). Furthermore, the genes associated with capsule synthesis and transport (Table S4), iron utilization and transport, and TonB-dependent receptor were significantly up- or downregulated in the C48-Δ*Pmorf0222* mutant strain (Table S5). The biofilm formation of the C48-Δ*Pmorf0222* mutant strain was detected *in vitro* (Fig. S8). These results indicated that *Pmorf0222* could regulate genes related to biofilm formation, capsule synthesis, and iron utilization in P. multocida strain C48-1.

## DISCUSSION

Although the main pathogenic serogroup and many virulence factors of P. multocida have been reported, the pathogenesis of capsule-type A P. multocida causing hemorrhagic sepsis and infectious pneumonia in animals is still not completely understood (4, 11, 18). Mobile genetic elements composed of mobile DNA regions encoding proteins are known as prophages, insertion sequences, transposons, or integrative conjugative elements. They are believed to be the major drivers of bacterial diversity, evolution, and even pathogenesis (19–22). The prophages contain genes encoding the virulence factors (e.g., toxins) critical for bacterial pathogenesis; these genes are found in almost all the sequenced bacterial genomes (23, 24). The discovery of PMT, cholera toxin, Corynebacterium diphtheriae toxin, and Clostridium botulinum toxin from phages supports that prophages are important for the evolution and virulence of many pathogens (23, 25–27). Whole-genome sequencing and comparative genomic analysis of P. multocida revealed that the genome of pathogenic strain C48-1 possesses one large unique region predicted to be a putative prophage similar to a *Mannheimia* lambda-like bacteriophage (4, 15). The *Pmorf0222* gene located in the specific prophage island of C48-1 encodes an HflK/C-like protein, which is present in only a few isolated P. multocida strains (see Table S6 in the supplemental material). The isolates whose genomes contain a gene with more than 95% homology with the *Pmorf0222* gene are P. multocida FCf15, P. multocida PMWG-4, P. multocida XG20, P. multocida HuN001, and P. multocida GS2020-X2, and most of them were derived from avian sources. These results suggested that the *Pmorf0222* gene might be closely related to the pathogenicity of avian P. multocida. Furthermore, the *Pmorf0222* gene deletion can strongly attenuate the virulence of C48-1 in mice. Further studies should characterize the other unknown proteins in the specific prophage island of C48-1.

Innate immune responses provide the first line of host defense against pathogenic infection by recognizing the pathogen-associated molecular patterns (PAMPs). As the pattern recognition receptors, TLRs are type I transmembrane glycoproteins characterized by the extracellular domains that mediate the recognition of their respective PAMP such as the outer membrane protein, lipoproteins, or LPS (28, 29). Additionally, TLRs possess a transmembrane domain and an intracellular domain that is homologous to that of the IL-1R and is known as the Toll/IL-1R domain. Studies have reported that TLR1 and TLR2 have extracellular domains, and TLR2 generally forms a heterodimer with TLR1 or TLR6 to establish a functional signaling complex for recognizing the bacterial diacylated or triacylated lipopeptide (29–35). IL-1β is a pleiotropic cytokine, which is a key mediator of inflammatory response and plays a crucial role in the host’s defense against bacterial infection (36, 37). The inflammasome is a multiprotein intracellular complex required for converting procaspase-1 to active caspase-1 and is responsible for the cleavage and release of mature IL-1β in macrophages (38). The NOD-like receptor (NLR)-family pyrin domain-containing 3 (NLRP3) inflammasome can sense various stimuli and generate an inflammasome complex with procaspase-1 and apoptosis-associated speck-like protein containing CARD (ASC). This leads to the maturation and release of caspase-1, which in turn cleaves the pro-IL-1β into its mature forms before release (39–41). Additionally, a previous study reported that the NLRP3 inflammasome plays an important role in caspase-1 activation and IL-1β secretion in macrophages exposed to P. multocida (13). In this study, rPM0222 induced the release of TNF-α and IL-1β through the TLR1/2–NF-κB/MAPK pathways. However, it remains unclear whether the TLR1/2–NF-κB and/or TLR1/2-MAPK signaling pathway is involved in the activation of the inflammasomes.

The P. multocida genome contains many genes encoding proteins involved in the biosynthesis, assembly, and secretion of virulence factors, such as capsule, adhesins, biofilm formation, proteins involved in iron metabolism, and OMVs (4, 42). The capsular hyaluronic acid of serogroup A of P. multocida is synthesized by the *phyBA-hyaEDCB-hexDCBA* gene cluster and is a glycosaminoglycan linked to lipids on the bacterial cell surface, and its structure is similar to that of the mammalian hyaluronic acid (43). The capsular hyaluronic acid is associated with the bactericidal complement sensitivity, adhesion, colonization, biofilm formation, and iron metabolism (17, 44). For example, the encapsulated strains of serogroup A of P. multocida are highly resistant to complement activity and survive actively in chicken serum (16). However, spontaneous mutants with defective capsules or strains treated with hyaluronidase are generally more sensitive to the bactericidal complement activity (45, 46). Additionally, previous reports have demonstrated that serogroup A of P. multocida can strongly adhere to host cells, and the process is mediated by the hyaluronic acid-binding cell surface transmembrane glycoprotein CD44 (17, 43). Recently, a study has confirmed that capsular hyaluronic acid production is inversely related to biofilm formation and possibly inhibits biofilm formation by blocking the surface proteins essential for adherence (47). As reported in this study, the expression of capsular hyaluronic acid in the C48-Δ*Pmorf0222* mutant strain significantly decreased at both RNA and protein levels, which might be the main method by which the *Pmorf0222* gene alters the phenotypes of P. multocida C48-1 strain. For example, *Pmorf0222* might enhance the adhesion to host cells and resistance to bactericidal complements in chicken serum and might interfere with iron metabolism and biofilm formation by regulating the production of hyaluronic acid; in turn, it might affect the virulence of serogroup A of P. multocida. However, the detailed mechanisms underlying the influence of *Pmorf0222* on the synthesis of hyaluronic acid are unclear.

Although some studies have reported that the pathogenicity of P. multocida is different in mice and chickens, mouse models of P. multocida infection have been established and used to explore the mechanism of pathogenesis of P. multocida infection (11, 18, 48–50). In this study, the pathogenicity experiment was performed in a mouse model; 200 CFU of the wild-type C48-1 strain was used as a control to evaluate the virulence of the mutant strain at an infection dose of 2 × 10^2^ to 2 × 10^7^ CFU. In our previous study, the minimum infection dose of the wild-type C48-1 strain causing 100% mortality in experimental chickens and mice was 20 CFU (data not published). However, further experimental exploration is still needed, assessing whether *Pmorf0222* affects the virulence of wild-type C48-1 and other strains in poultry.

In this study, a new virulence factor, *Pmorf0222*, from the specific prophage island of the pathogenic strain C48-1 of P. multocida was identified and characterized. PM0222 protein secreted by OMVs of P. multocida could induce the secretion of TNF-α and IL-1β via the TLR1/2–NF-κB/MAPK and inflammasome signaling pathways. These results provided a new perspective for understanding the role of the SPFH superfamily HflK/C-like proteins in the pathogenicity of P. multocida and secretion of proinflammatory cytokines by murine peritoneal exudate macrophages.

## MATERIALS AND METHODS

### Bacterial strains, cells, antibodies, and animals.

The pathogenic strain C48-1 (A:1) of P. multocida was isolated from chicken with fowl cholera in Jiangsu, China, in 1953 and was stored at our laboratory. The murine peritoneal exudate macrophages were isolated from C57BL/6N mice. The DF-1 and HD11 cells were provided by our laboratory. The antibodies used were as follows: antibodies against ERK1/2, p38, JNK, p65, p-ERK1/2, p-p38, p-JNK, and p-p65 (Cell Signaling Technology, USA); procaspase-1 and IL-1β (Abcam, England); GSDMD or GSDMD(-N), ASC, and caspase-1 p20 (Affinity, USA); NLRP3 (Proteintech, China); TLR1, TLR2, and TLR4 and IgG isotype-matched control antibodies (Proteintech, China); and β-actin (Yeasen, China). The NF-κB (BAY11-7082), JNK (SP600125), p38MAPK (SB203580), and ERK (U0126) inhibitors were purchased from Sigma-Aldrich (USA). The IRDye 800CW-conjugated anti-rabbit IgG or anti-mouse IgG antibodies were purchased from Li-Cor Biotechnology (USA). In this study, 5- to 8-week-old C57BL/6N mice were purchased from Liaoning Changsheng Biotechnology (Liaoning, China), and SPF chicken serum was provided by the National Engineering Research Center of Veterinary Biologics Corp. (Harbin, China).

### Construction of the mutant strain and complemented strain.

The upstream (801-bp) and downstream (743-bp) sequences of *Pmorf0222* (GenBank accession number OAZ06802.1) in the C48-1 strain were amplified using PCR using the primers Pm0222-BamHI-U1/Pm222-RH-U2 and Pm0222-RH-D3/Pm0222-SacI-D4, respectively. The upstream and downstream sequences of *Pmorf0222* were linked with the primers Pm0222-SacI-U1/Pm0222-BamHI-D4 by overlap PCR. The homologous sequences and temperature-sensitive plasmids of pSHK5(TS)-*Ng*Ago, capable of replicating autonomously at 30°C but not at 42°C, were digested using SacI and BamHI. Further, the recombinant plasmid pSHK5(TS)-*Ng*Ago-Pmorf0222UD was constructed.

For producing *Pmorf0222*-deletion mutant strain, the electrocompetent cells of the C48-1 strain were prepared using the method described previously (51). Briefly, the recombinant plasmid pSHK5(TS)-*Ng*Ago-Pmorf0222UD was electrotransformed (at 2.5 kV, 500 Ω, and 25 μF) into prepared competent cells of the strain C48-1. These recombinant cells were resuspended in 800 μL of brain heart infusion (BHI; Difco Laboratories, USA) medium and recovered at 28°C for 3 h. Further, 200 μL of the suspension was spread onto BHI agar containing 100 μg/mL kanamycin and incubated for 48 to 72 h at 28°C. To confirm the knockout of *Pmorf0222*, the bacterial clones were subjected to PCR using the primers Pm0222-ID-F/R complementary to the genome sequences at the left of the upstream and right of the downstream of *Pmorf0222*. *Pmorf0222* deletion was confirmed using Western blotting and DNA sequencing, and the mutant strain was named C48*-ΔPmorf0222*. At the same time, *Pmorf0222* gene expression cassettes comprising a 255-bp upstream segment, a 42-bp downstream segment, and 906-bp *Pmorf0222* were amplified from the genomic DNA of the wild-type strain using PCR using the primers Pm0222-F/R. Further, they were cloned into the shuttle plasmid pAL99 for constructing the recombinant plasmid pAL99-Pmorf0222. Further, the recombined plasmid pAL99-Pmorf0222 was electrotransformed into the C48-Δ*Pmorf0222* mutant strain under the same conditions as described above. The transformed cells were cultured on BHI agar containing 100 μg/mL kanamycin and incubated at 37°C, generating the corresponding complemented strain C48-Δ*Pmorf0222^C^*. The bacterial strains and plasmids are listed in Table 1. The sequences of the primers synthesized by Comate (Jilin, China) are listed in Table S2 in the supplemental material.

**TABLE 1:** Bacterial strains and plasmids used in this study^*a*^

Strain or plasmid	Description	Source or reference
Strains		
E. coli DH5α	Cloning host for recombinant vector	TaKaRa
E. coli TB1	Expression host for recombinant protein	TaKaRa
P. multocida C48-1	Serotype A:1, chicken; Jiangsu, China	CVCC, China
C48-Δ*Pmorf0222*	C48-1 strain *Pmorf0222* gene mutant	This study
C48-Δ*Pmorf0222^C^*	C48-Δ*Pmorf0222* containing pAL99-*Pmorf0222*	This study
Plasmids		
pMAL-c5x-His	Expression vector; Amp^r^	New England BioLabs
pSHK5(TS)-*Ng*Ago	*Ng*Ago and RBS gene insert between the Kan promoter and Kan^r^ gene of temperature-sensitive plasmid pSHK5(TS), can express *Ng*Ago protein	Gifted by Anding Zhang
pSHK5(TS)-*Ng*Ago-Pmorf0222UD	pSHK5(TS)-*Ng*Ago containing up and down arms of *Pmorf0222*, constructing *Pmorf0222* gene deletion mutant	This study
pAL99	Shuttle plasmid in E. coli and P. multocida	Gifted by Ben Adler
pAL99-Pmorf0222	pAL99 containing *Pmorf0222* gene expression cassettes	This study

a Amp^r^, ampicillin resistant; Kan^r^, kanamycin resistant; RBS, ribosome binding site; CVCC, China Veterinary Culture Collection.

### Virulence assessment in mice.

To assess the role of *Pmorf0222* in the virulence of P. multocida, C57BL/6N mice (*n *= 7 per group) were infected with the wild-type strain, mutant strain, or complemented strain via intraperitoneal injection at a dose of 2 × 10^2^ to 2 × 10^7^ CFU. The virulence levels of the wild-type strain, mutant strain, or complemented strain were compared using the survival curve of the mice and mortality on the 7th day postinfection. The liver and spleen tissues of the mice infected with equal doses (2 × 10^3^ CFU) for 6 h in the three groups after injection were collected to determine the bacterial load (*n *= 10 per group). All the animal experiments were performed according to the animal protocols approved by the Subcommittee on Research Animal Care of Harbin Veterinary Research Institute (HVRI) and the Chinese Academy of Agricultural Sciences. The Institutional Animal Care and Use Committee (IACUC) number is HVRI-IACUC-2019-214, and the animal experiment number is 210201-04.

### Evaluation of biofilm formation.

The ability of biofilm formation was quantified using the crystal violet (CV) method (47). Briefly, 200 μL of the bacterial inoculum with an optical density at 600 nm (OD_600_) of 0.4 to 0.6 was transferred to a polystyrene 96-well microtiter plate in triplicates (Costar, USA) and cultured for 12 to 42 h. Further, the bacterial culture was washed three times with sterile phosphate-buffered saline (PBS) and incubated with 200 μL of 0.1% CV at 20 to 25°C for 10 min. The CV solution was discarded, and the plate was rinsed thoroughly with sterile PBS. Subsequently, the CV was solubilized using 200 μL ethanol, and the OD_630_ of the dye solutions was measured using a Gen5 microplate reader (BioTek, USA).

### Determination of the capsular hyaluronic acid content.

The capsular hyaluronic acid content was quantified using the method described previously (16). Each group of strains grown until the logarithmic phase was washed three times with ice-cold sterile distilled water and suspended in 1 mL sterile distilled water. To this, 1 mL chloroform was added, followed by vortexing the mixture for 10 min to release the capsule in the aqueous phase. After centrifuging the mixture at 1,000 × *g*, the amount of hyaluronic acid in the aqueous phase was quantified by measuring OD_640_ after adding 2 mL of a staining buffer {prepared by adding 10 mg 1-ethyl-2-[3-(1-ethylnaphtho-[1,2-d]thiazolin-2-ylidene)-2-methylpropenyl]naphtho-[1,2-d]thiazolium bromide (Sigma-Aldrich, USA) to 30 mL glacial acetic acid in 50 mL of 50% formamide}. The absorbance was measured, and concentrations were calculated based on the standard curve plotted with the known concentrations of hyaluronic acid (52).

### Assessment of sensitivity to chicken serum.

To determine the serum sensitivity of the P. multocida strains and control strain E. coli DH5α, approximately 10^5^ CFU of strains from each group in the logarithmic phase was incubated with 8-week-old SPF chicken serum and heat-inactivated (30 min at 60°C) SPF chicken serum for 3 h at 37°C. The bactericidal activity of serum was calculated as the survival ratio. The assays were performed in triplicates for the P. multocida and control E. coli DH5α strains. The SPF chicken serum was provided by the National Engineering Research Center of Veterinary Biologics Corp. (Harbin, China).

### Cell-based assays.

To confirm the ability of the mutant strain to adhere to the host cells, DF-1 and HD11 cells were seeded in 12-well plates and grown in Dulbecco’s modified Eagle’s medium (DMEM) containing 10% fetal bovine serum (FBS) (Sigma-Aldrich, USA) at 37°C with 5% CO_2_ for 24 h. The wild-type strain, mutant strain, or complemented strain in logarithmic phase was washed twice with DMEM and added to the wells in triplicates at a multiplicity of infection (MOI) of 10. The bacterium and cell mixture was centrifuged at 400 × *g* for 5 min and incubated at 37°C for 2 h. The infected cells were thoroughly washed five times with sterile PBS and lysed with 0.2% Triton X-100. The number of adherent bacteria was calculated by plating them on BHI agar plates incubated at 37°C for 12 to 18 h.

At the same time, the role of *Pmorf0222* in the virulence of P. multocida was confirmed by incubating the wild-type strain, mutant strain, and complemented strain with the murine peritoneal exudate macrophages for 6 h at an MOI of 10. The levels of IL-1β and TNF-α in the cell supernatant were determined using the mouse IL-1β and TNF-α enzyme-linked immunosorbent assay (ELISA) kits, respectively.

### Purification of the recombinant PM0222 protein and OMVs.

*Pmorf0222* gene fragments were digested with the primers Pm222-EF/R (Table S2) and were inserted into the vector pMAL-c5x-His to generate pMAL-c5x-*Pmorf0222*-His. Further, they were used to transform into E. coli TB1 competent cells for expression with 0.5 mM isopropyl-β-d-1-thiogalactopyranoside (Tiangen, China) at 16°C. The purified recombinant (maltose-binding protein, MBP)-PM0222 fusion proteins were subjected to dialysis at least twice in the 100× volume of factor Xa reaction buffer (20 mM Tris, 100 mM NaCl, 2 mM CaCl_2_; pH 7.5) at 4°C for 5 h. After dialysis, the recombinant MBP-PM0222 fusion proteins were digested for 8 h at 23°C using the factor Xa protease (New England BioLabs, USA) according to the manufacturer’s protocol. The factor Xa-digested protein was purified separately using the amylose resin (New England BioLabs, USA) column and His-Sep nickel-nitrilotriacetic acid (Ni-NTA) agarose resin (Yeasen, China) affinity column to collect the MBP-removed rPM0222 proteins containing the C-terminal polyhistidine (6×His) tag. Further, rPM0222 was subjected to sodium dodecyl-sulfate polyacrylamide gel electrophoresis (SDS-PAGE) and Western blotting, and the protein was quantified using the bicinchoninic acid (BCA) kit (Thermo, USA) and stored at −80°C until use. In Western blot analysis, the signals of the PM0222 protein expressed by different wild-type strain C48-1 CFU values (OD_600_ of approximately 0.6) were quantified using ImageJ software (National Institutes of Health, USA). The fitting curve was established based on various C48-1 CFU and intensity values of Western blot signals of PM0222 protein expressed by various C48-1 CFU values to estimate the CFU equivalent for the amount of rPM0222 protein.

The OMVs were isolated using the method described previously (42). The wild-type strain and mutant strain were grown in 1 L BHI at 37°C for 24 h. The bacterial cells were precipitated by centrifugation at 12,000 × *g* for 10 min at 4°C. Further, the residual cells in the culture supernatants were removed by passing the solution through a membrane with an 0.22-μm pore size. The OMVs were subjected to ultracentrifugation at 150,000 × *g* for 3 h at 4°C. Equal amounts of proteins (10 μg) from each group of strains were subjected to Western blotting using the anti-rPM0222 mouse serum as the primary antibody.

Every 2 weeks, the antiserum was prepared by subcutaneously immunizing mice three times with rPM0222 supplemented with an equal volume of Freund’s incomplete adjuvant. The serum was collected 7 days after the last immunization, and the antiserum titers were determined using ELISA.

### Isolation and culture of murine peritoneal exudate macrophages.

The murine peritoneal exudate macrophages were isolated using the method described previously (12). Briefly, C57BL/6N mice were injected intraperitoneally with 2 mL 4% thioglycolate broth (Sigma-Aldrich, USA), and the murine peritoneal exudate macrophages were collected after 48 to 72 h. The murine peritoneal exudate macrophages were suspended in RPMI 1640 medium (Sigma-Aldrich, USA) containing 10% FBS and counted using a hemocytometer. They were seeded into 24-well microplates at a density of 3 × 10^5^ cells/well and incubated at 37°C with 5% CO_2_. The nonadherent cells were removed by rinsing the wells with sterile PBS after 2 h.

### RNA sequencing.

The cDNA libraries from the three replicates of all samples were constructed and sequenced by Gene Denovo, Ltd. (Guangzhou, China), on the Illumina sequencing platform (HiSeq 6000), producing 150-bp single-end reads. All the clean reads were aligned to the reference genome (GCA_004286945.1) of the P. multocida C48-1 strain using Bowtie2 software (version 2.2.8). The fragment-per-kilobase-per-million values for the genes were calculated using the edgeR package (http://www.r-project.org/). Based on the threshold of log_2_ fold change of ≥1 and false-discovery rate-adjusted *P* (*q* value) value of <0.05, the DEGs were confirmed using the edgeR package by comparison with the annotation of the reference transcripts. All the DEGs were annotated to the Gene Ontology and KEGG pathway databases. *q* values were corrected using <0.05 as the threshold.

### Cytokine ELISAs.

The murine peritoneal exudate macrophages were seeded into 24-well plates at a density of 3 × 10^5^ cells/well and stimulated for various times (3, 6, 9, or 12 h) or with various doses (1, 3, 5, or 7 μg/mL) using rPM0222. A purified aliquot of rPM0222 was filtered through a 0.22-μm filter (Merck, USA) and incubated with polymyxin B (Thermo, USA), which acts as an LPS inhibitor to eliminate the potential LPS contamination. To determine whether the proinflammatory cytokine secretion from the murine peritoneal exudate macrophages was specifically induced by rPM0222, rPM0222 was preincubated for 1 h at 37°C with the anti-rPM0222 serum or control serum. To determine whether rPM0222 is responsible for the activation of MAPK and NF-κB, the murine peritoneal exudate macrophages were pretreated for 1 h at 37°C with specific inhibitors of NF-κB (BAY11-7082), JNK (SP600125), p38MAPK (SB203580), and ERK (U0126) before their incubation with rPM0222. To determine whether rPM0222 is responsible for the activation of TLR signaling, the murine peritoneal exudate macrophages were pretreated for 1 h at 37°C with antibodies against TLR1 (5 μg/mL), TLR2 (5 μg/mL), and TLR4 (5 μg/mL) or an IgG isotype-matched control antibody (5 μg/mL) before the incubation with rPM0222. Further, the cell culture supernatants were collected, and the concentrations of IL-1β and TNF-α in them were quantified using ELISA kits (Neobioscience, China).

### qRT-PCR.

The cellular and bacterial total RNA was extracted using the RNeasy Plus minikit (Qiagen, Germany) according to the manufacturer’s protocol. The potential contamination of DNA was removed during RNA extraction. cDNA was synthesized using a PCR protocol as follows: 25°C for 10 min, followed by 42°C for 30 min and 85°C for 5 min using the 5× All In One RT MasterMix reagent kit (ABM, Canada). Quantitative real-time PCR (qRT-PCR) was performed using the EvaGreen 2× qPCR MasterMix-No Dye reagents (ABM, Canada) according to the manufacturer’s instructions and on a QuantStudio 5 real-time PCR system (Applied Biosystems, USA). The sequences of primers used in qRT-PCR are listed in Table S2.

### Western blot analysis.

The murine peritoneal exudate macrophages were seeded into 24-well plates at a density of 3 × 10^5^ cells/well. As described previously, a purified aliquot of rPM0222 was filtered and potential LPS contamination was removed by treatment with LPS inhibitor polymyxin B (30 μg/mL). The stimulated cells were lysed using radioimmunoprecipitation assay buffer (Beyotime, China) for various times or with various doses with rPM0222 on ice for 1 h. The supernatant was collected by centrifugation at 12,000 × *g* for 10 min at 4°C, and the protein was quantified using the BCA protein assay kit. Equal amounts of protein were subjected to SDS-PAGE and transferred onto nitrocellulose membranes (Merck, USA). After being blocked with 5% skimmed milk (Difco Laboratories, USA) in Tris-buffered saline with Tween 20 for 2 h at 20 to 25°C, the membranes were incubated with primary antibodies for 2 h at 20 to 25°C. The primary antibodies were as follows: against p65, ERK1/2, p38, p-p65 (S536), p-ERK1/2 (T202/Y204), p-p38 (T180/Y182), p-JNK (T183/Y185), NLRP3, procaspase-1, caspase-1 p20, ASC, pro-IL-1β, mature form of IL-1β, and β-actin. Subsequently, the membranes were incubated for 1 h with IRDye 800CW-conjugated anti-rabbit IgG or anti-mouse IgG antibodies, and the signals were detected using ODyssay Clx (Li-Cor Biotechnology, USA). The signal intensity values of p-p65, p-ERK1/2, p-p38, p-JNK, and β-actin in Western blotting were quantified using ImageJ software (National Institutes of Health, USA).

### Confocal microscopy.

Bacterial biofilm formation was observed using CLSM after incubation of the bacterial strains. First, 1 mL of the bacterial inoculum with an OD_600_ of 0.4 to 0.6 was transferred to a glass-bottom cell culture dish (Biosharp, China) and cultured at 37°C for 36 h. Further, the wells were washed three times with water to remove loosely attached bacterial cells. The nuclei were stained using 4′,6-diamidino-2-phenylindole (DAPI; Invitrogen, USA). The plate was incubated for 15 min in the dark and washed with sterile PBS. The wells were observed under fluorescence mode using a CLSM LSM880 microscope (Leica Microsystems, Germany).

For bacterial adhesion assay, the bacteria were stained using carboxyfluorescein succinimidyl amino ester (CFSE; Invitrogen, USA) according to the manufacturer’s protocol. HD11 cells were seeded into a glass-bottom cell culture dish and infected with the stained bacteria at an MOI of 10 for 2 h. The cells were washed with sterile PBS and fixed with 4% paraformaldehyde for 10 min at 20 to 25°C, permeabilized with 0.1% Triton X-100 for 10 min, and blocked with 1% bovine serum albumin (BSA) for 1 h at 20 to 25°C. Further, the stained bacteria were incubated with rabbit anti-P. multocida C48-1 serum for 24 h at 4°C and further labeled with goat anti-rabbit IgG(H+L) Alexa Fluor 633 conjugate for 1 h at 20 to 25°C. The nuclei were stained with DAPI for 10 min at room temperature, further thoroughly rinsed three times with sterile PBS, and visualized under fluorescence mode using a CLSM LSM880 microscope. Every 2 weeks, anti-P. multocida C48-1 serum from rabbits was prepared by subcutaneously immunizing rabbits three times with inactivated P. multocida C48-1 strains supplemented with an equal volume of adjuvant. The serum was collected 7 days after the last immunization, and the antiserum titers were determined using ELISA.

For analyzing the activity of inflammasomes, the cells were seeded on a glass-bottom cell culture plate at a density of 1 × 10^6^ cells/well and incubated with rPM0222 or LPS at 37°C with 5% CO_2_ for 6 h. The cells were washed with sterile PBS, fixed with 4% paraformaldehyde for 10 min at 20 to 25°C, permeabilized with 0.1% Triton X-100 for 10 min, and blocked using 1% BSA for 1 h at 20 to 25°C. The cells were incubated with rabbit anti-NLRP3 or rat anti-ASC antibody for 24 h at 4°C, followed by incubation with goat anti-rabbit IgG(H+L) and goat anti-rat IgG(H+L) Alexa Fluor 633 (Thermo, USA) for 1 h at 20 to 25°C. The nuclei were stained with DAPI for 10 min at 20 to 25°C, thoroughly washed three times with sterile PBS, and visualized under fluorescent mode using a CLSM LSM880 microscope.

### Statistical analysis.

Data obtained from experiments repeated at least thrice were presented as the mean ± standard deviation. GraphPad Prism version 6.0 (GraphPad Software, USA) was used for statistical analysis. For the data in the Gaussian distribution, two groups were compared using unpaired *t* tests if the data exhibited equal variance or using an unpaired *t* test using Welch’s correction if the data exhibited unequal variance. If the data were not normally distributed, they were analyzed using a nonparametric test (Mann-Whitney *U* test). The Gaussian distribution of the data was analyzed using the D’Agostino-Pearson omnibus normality test and the Kolmogorov-Smirnov test. The variance of data was analyzed using the Brown-Forsythe test. A *P* value of <0.05 was considered significant.

### Data availability.

The transcriptome analysis data sets generated in this study can be found at https://www.ncbi.nlm.nih.gov/sra, and the accessions numbers for these data sets are SRR16314803, SRR16314807, SRR16314806, SRR16314805, SRR16314804, and SRR16314802.
