# Supplementary material for: Pmorf0222, a Virulence Factor in Pasteurella multocida, Activates Nuclear Factor Kappa B and Mitogen-Activated Protein Kinase via Toll-Like Receptor 1/2
Source: Infect Immun. 2022 Dec 21;91(1):e00193-22. doi: 10.1128/iai.00193-22 (PMC9872710; doi:10.1128/iai.00193-22)
Supplement: Supplemental file 2 — Fig. S1 to S8. Download iai.00193-22-s0002.pdf, PDF file, 1.1 MB [file iai.00193-22-s0002.pdf]

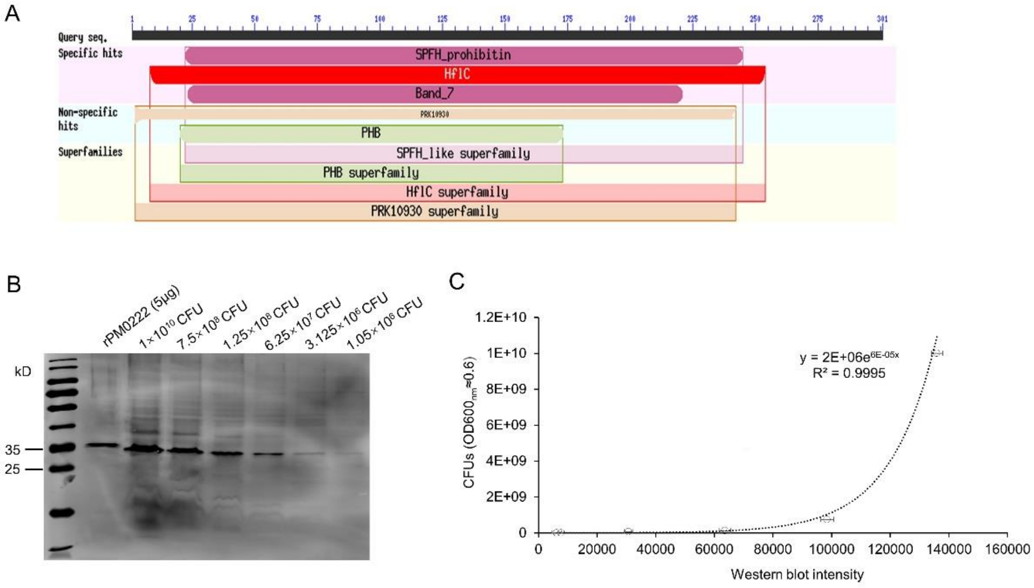

FIG S1 (A) Conserved domain prediction of the *P. multocida C48-1* PM0222 protein. (B) Western blot signals of rPM0222 and PM0222 proteins expressed by various *C48-1* CFUs ($OD_{600\ nm}$ of approximately 0.6). (C) Fitting curves based on various different *C48-1* CFUs and intensity values of western blot signals of PM0222 protein expressed by different *C48-1* CFUs. The experiments were performed at least three times.

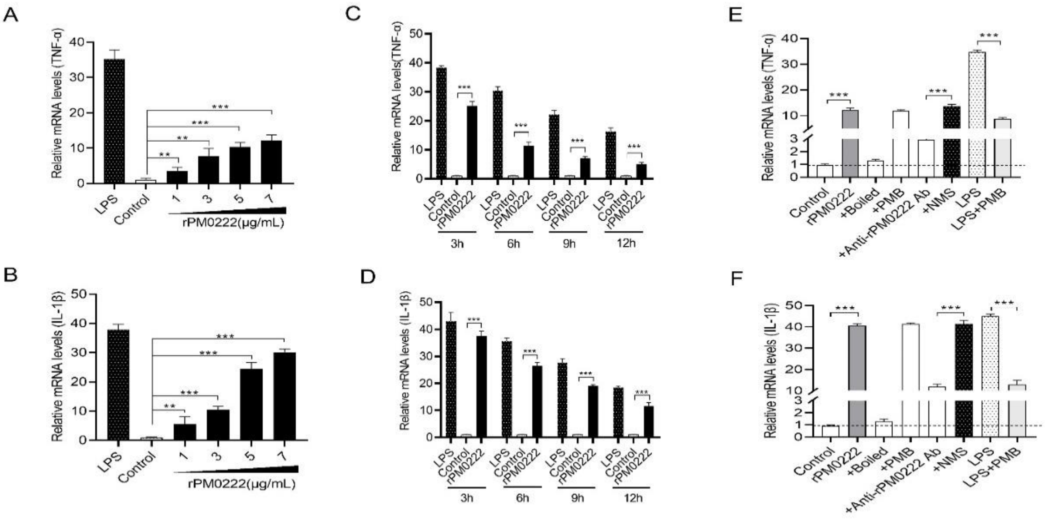

FIG S2 The murine peritoneal exudate macrophages were incubated with rPM0222 protein (1, 3, 5, or 7 μg/mL) for 6 h, and relative mRNA levels of *TNF-α* (A) and *IL-1β* (B) were calculated and expressed as fold change relative to the control group. The murine peritoneal exudate macrophages were incubated with 5 μg/mL rPM0222, and relative mRNA levels of *TNF-α* (C) and *IL-1β* (D) were calculated and expressed as fold change relative to the control group at 3, 6, 9, or 12 h. The murine peritoneal exudate macrophages were incubated with 5 μg/mL rPM0222 for 6 h, and relative mRNA levels of *TNF-α* (E) and *IL-1β* (F) were calculated and expressed as fold change relative to the control group. rPM0222 was pretreated with polymyxin B (+PMB) and boiled (+boiled) at 100 °C for 10 min to confirm whether the activation of murine peritoneal exudate macrophages was because of rPM0222 and not LPS. LPS (1 μg/mL) was used as a positive control. LPS (1 μg/mL) was pretreated with polymyxin B to confirm the action of PMB (LPS+PMB). To confirm that rPM0222 specifically promoted the secretion of pro-inflammatory cytokine, the proteins (5 μg/mL) were pretreated with the anti-rPM0222 sera (+anti-rPM0222 Ab, 1:500) or control mouse sera (+NMS, 1:500). Data are expressed as the mean ± SD from three independent experiments. **$P < 0.01$, ***$P < 0.001$. The experiments were performed at least three times.

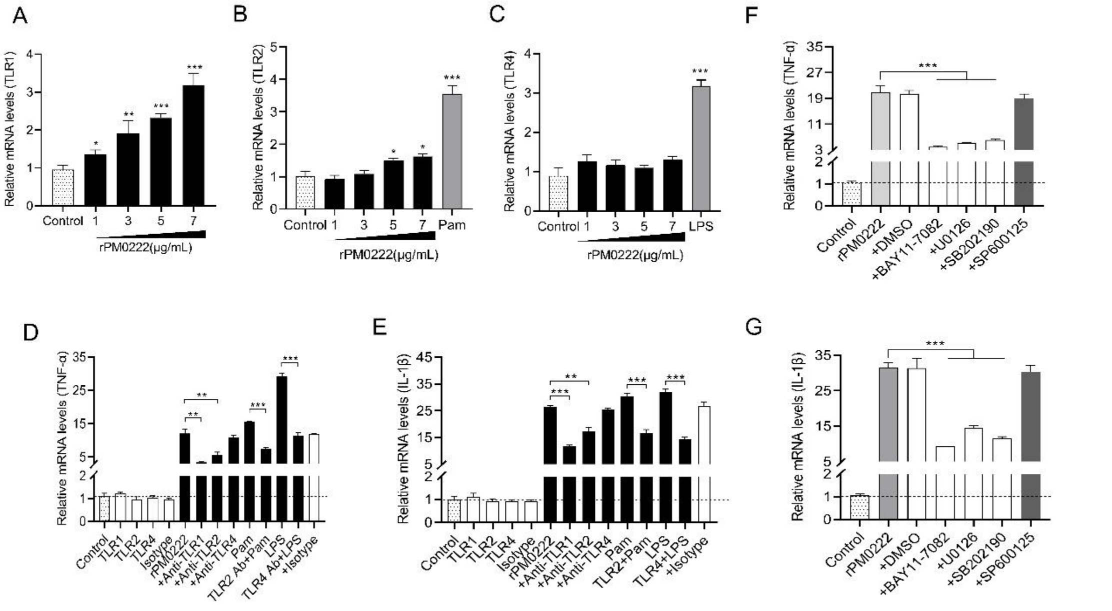

FIG S3 The murine peritoneal exudate macrophages were cultured for 6 h in the presence of various concentrations of rPM0222. The relative mRNA levels of *TLR1* (A), *TLR2* (B), and *TLR4* (C) were calculated and expressed as fold change relative to the control group. The cells were pretreated separately with 5 μg/mL each of anti-TLR1 (+anti-TLR1), anti-TLR2 (+anti-TLR2), anti-TLR4 (+anti-TLR4), or IgG isotype-matched control group antibody (+Isotype) for 1 h and stimulated with rPM0222 (5 μg/mL) for 6 h. The relative mRNA levels of *TNF-α* (D) and *IL-1β* (E) were calculated and expressed as fold change relative to the control group. The murine peritoneal exudate macrophages were pretreated with U0126 (+U0126; 20 μM), SB203580 (+SB202190; 20 μM), SP600125 (+SP600125; 20 μM), BAY11-7082 (+BAY11-7082; 20 μM), or dimethyl sulfoxide (0.01%) (+DMSO) for 1 h and stimulated with rPM0222 (5 μg/mL) for 6 h. The relative mRNA levels of *TNF-α* (F) and *IL-1β* (G) were calculated and expressed as fold change relative to the control group. Data are expressed as the mean ± SD from three independent experiments. **P* < 0.05, ***P* < 0.01, ****P* < 0.001. The experiments were performed at least three times.

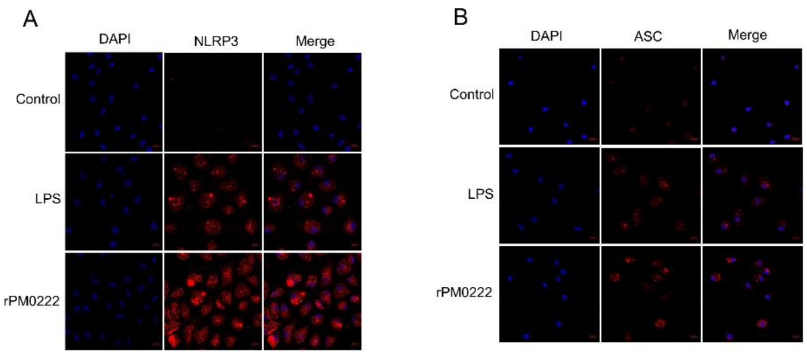

FIG S4 The murine peritoneal exudate macrophages were incubated with 5 μg/mL rPM0222 for 6 h. Representative images are shown for NLRP3 (A) or ASC (B) with nuclear co-localization. Red, NLRP3 or ASC; blue, nucleus. The experiments were performed at least three times.

A

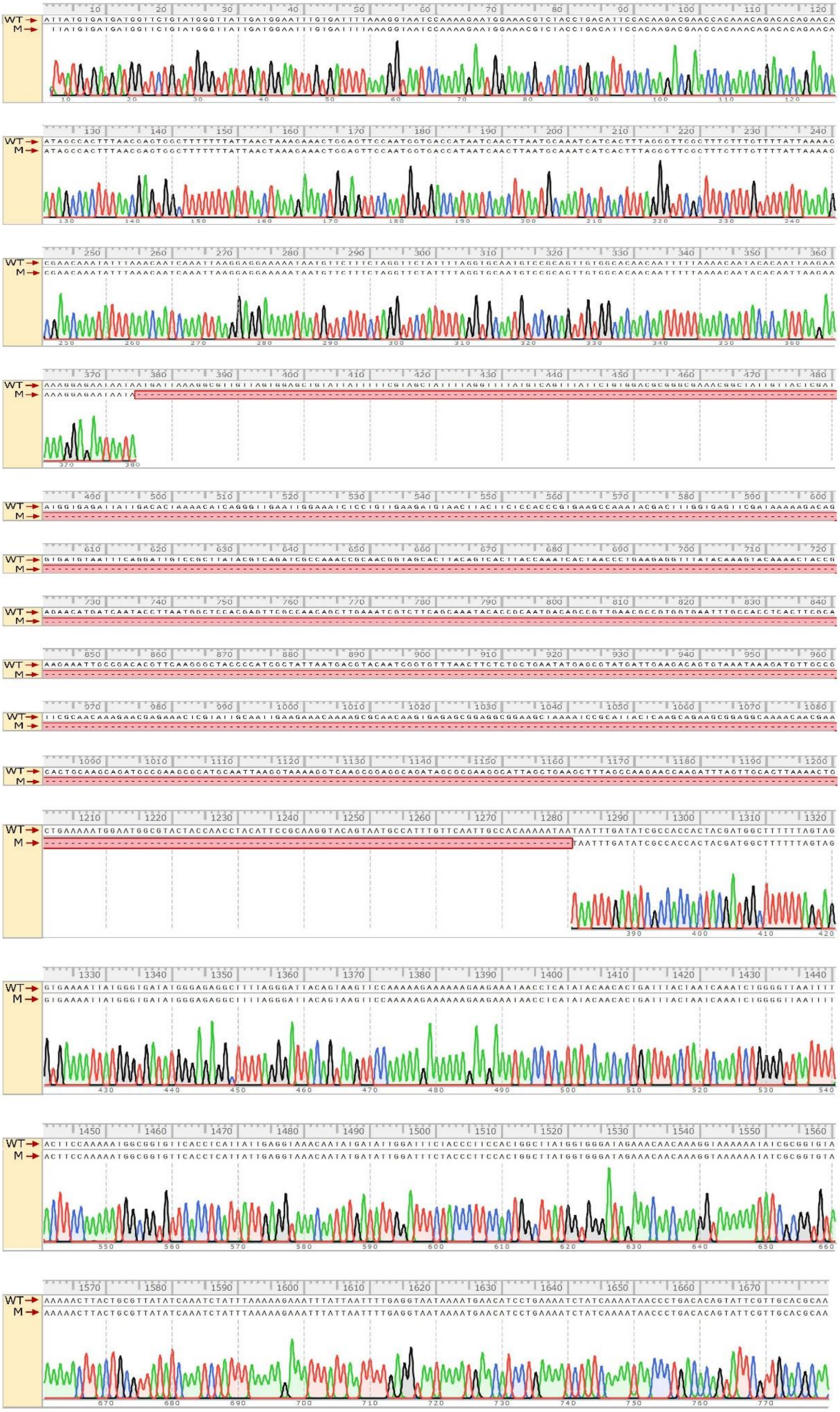

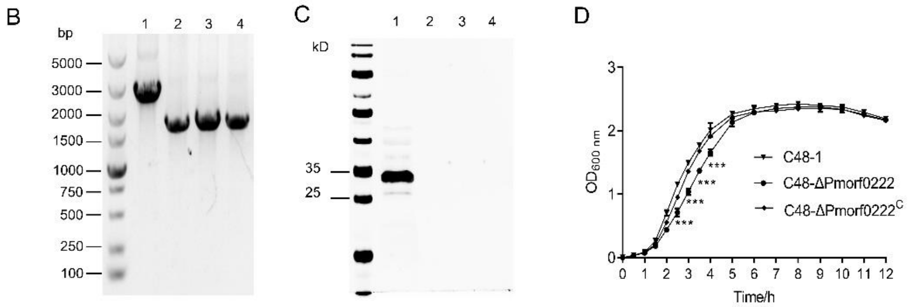

FIG S5 Identification of *C48-ΔPmorf0222* mutant strain. (A) Identification of the mutant strain using DNA sequencing after 20 rounds of subculture. WT, DNA sequence of wild type *C48-1* strain; M, DNA sequencing of mutant. *pmorf0222* gene sequence of mutant is given in red box. Lane 1, identification of the wild-type strain using PCR (B) or western blot analysis (C). Lane 2, 3, and 4, identification of the mutant strain at the primary culture, 10th, and 20th generation subculture using PCR (B) or western blot (C). (D) Growth curves of the wild-type strain, mutant strain, and complemented strain based on the OD_{600} nm reading. Data are expressed as the mean ± SD from three independent experiments. ***$P < 0.001$. The experiments were performed at least three times.

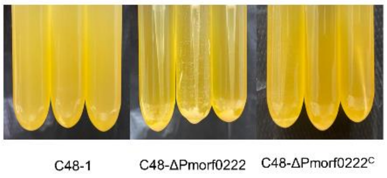

FIG S6 Representative images are shown for the wild type strain, mutant strain, and complemented strain after standing for the same time.

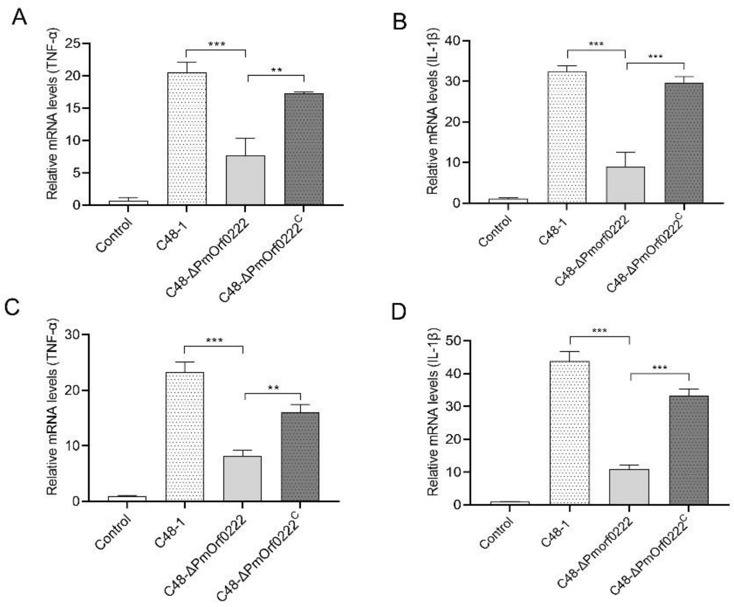

FIG S7 Induction of *TNF-α* and *IL-1β* from HD11 cells (A and B) and murine peritoneal exudate macrophages (C and D) by the wild type strain, mutant strain, and complemented strain at an M.O.I. of 10 for 6 h. The relative mRNA levels of *TNF-α* and *IL-1β* were calculated and expressed as fold change relative to the control group. Data are expressed as the mean ± SD from three independent experiments. $^{**}P < 0.01$, $^{***}P < 0.001$. The experiments were performed at least three times.

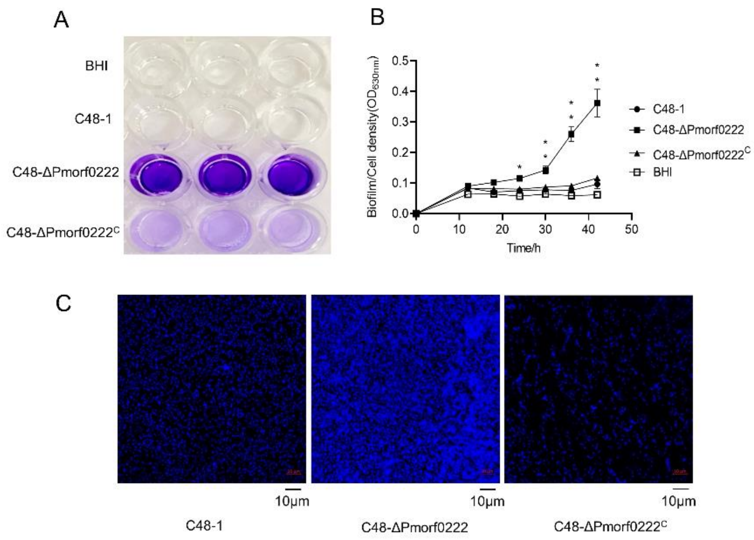

FIG S8 Detection of the biofilm formation. (A) Biofilm formation of the wild type strain, mutant strain, and complemented strain on a polystyrene microtiter plate at 36 h. (B) The quantitative analysis of biofilm formation of the wild type strain, mutant strain, and complemented strain after 12, 18, 24, 30, 36, and 42 h of incubation. (C) The biofilm formation of the wild type strain, mutant strain, and complemented strain were observed using CSLM after 36 h of incubation. The bacteria were stained with CFSE, and nuclei were stained with DAPI. Data are expressed as the mean ± SD from three independent experiments. $*P < 0.05$, $**P < 0.01$. The experiments were performed at least three times.
